# Relationship between Handgrip Strength and Incident Diabetes in Korean Adults According to Gender: A Population-Based Prospective Cohort Study

**DOI:** 10.3390/jcm13020627

**Published:** 2024-01-22

**Authors:** Sung-Bum Lee, Min-Kyeung Jo, Ji-Eun Moon, Hui-Jeong Lee, Jong-Koo Kim

**Affiliations:** 1 Department of Family Medicine, Soonchunhyang University Bucheon Hospital, Bucheon 22972, Republic of Korea; sblee@schmc.ac.kr (S.-B.L.); 112659@schmc.ac.kr (M.-K.J.); lhj@schmc.ac.kr (H.-J.L.); 2 Department of Medicine, Graduate School, Yonsei University Wonju College of Medicine, Wonju 26426, Republic of Korea; 3 Department of Biostatistics, Clinical Trial Centre, Soonchunhyang University Bucheon Hospital, Bucheon 14584, Republic of Korea; moon6188@schmc.ac.kr; 4 Department of Family Medicine, Yonsei University Wonju College of Medicine, Wonju 26426, Republic of Korea; 5 Institute of Global Health Care and Development, Wonju 26426, Republic of Korea

**Keywords:** handgrip strength, sarcopenia, diabetes, sex difference

## Abstract

(1) Background: Diabetes mellitus (DM) is a well-known disease that causes comorbidities such as chronic kidney disease (CKD) and cardiovascular disease. Therefore, it is necessary to develop diagnostic tools to prevent DM. Handgrip strength, a known diagnostic tool for sarcopenia, is a predictor of several diseases. However, the value of handgrip strength as an indicator of incident DM in Asian populations remains unknown. This study aimed to identify the relationship between handgrip strength and incidence of DM in Korean adults according to sex. (2) Methods: A total of 173,195 participants registered in a nationwide cohort were included in this study. After applying the exclusion criteria, 33,326 participants remained. DM occurred in 1473 individuals during the follow-up period (mean follow-up period, 4.1 years). To reduce the impact of body size, the study population was subdivided into quartiles of relative handgrip strength, defined as absolute handgrip strength divided by body mass index. Multivariate Cox regression analysis revealed that the relative handgrip strength was inversely associated with new-onset DM. (3) Results: Compared with the lowest quartile (Q1), the hazard ratios (HRs) [95% confidence intervals (CIs)] for new-onset DM for the highest quartiles (Q4) was 0.60 (0.43–0.84) in men and 0.72 (0.52–0.99) in women after adjusting for confounding factors. The incidence of DM decreased with the increase in the relative handgrip strength. These inverse relationships were statistically more significant in men than in women. (4) Conclusions: This novel study revealed that relative handgrip strength is related to incident DM in both men and women. Relative handgrip strength can be used as a practical tool to prevent DM. Regular measurement of handgrip strength can be used to detect DM.

## 1. Introduction

The number of adults diagnosed with diabetes worldwide was approximately 285 million (6.4%) in 2010. This number is predicted to rise to roughly 439 million (7.7%) by 2030 [1]. Individuals with diabetes are more susceptible to severe life-threatening health issues, which result in higher medical care costs, reduced quality of life, and increased mortality [2]. Therefore, it is important to diagnose and treat diabetes to prevent its complications. In addition, this would be useful in predicting and preventing diabetes progression.

Sarcopenia is characterized as a decline in muscle mass and strength associated with aging [3]. The diagnostic criteria for sarcopenia have not yet been unified. Therefore, its prevalence ranges between 9.9 and 40.4% depending on the diagnostic criteria used [4]. Among the several methods used to measure sarcopenia, determining handgrip strength (HGS) is a simple and easy technique that can be used in clinics [5]. Moreover, studies have suggested that relative handgrip strength (RGS), defined as handgrip strength divided by body mass index (BMI), is a more practical predictor of sarcopenia than HGS [6,7].

Previous studies have mainly focused on the relationship between HGS and the prevalence of diabetes. Mauro et al. demonstrated an inverse association between RGS and the prevalence of diabetes in older Italian women [8]. However, the study participants were limited to obese women with an average age of approximately 70 years, and the number of participants was relatively low (approximately 600) [8]. Lee et al. reported an inverse association between RGS and prevalence of diabetes mellitus (DM) in a study targeting a Korean population [9]. The number of participants was approximately 20,000, which gave the study the advantage of being a large-group study. However, since it was a cross-sectional study, the relationship between RGS and the incidence of DM could not be determined [9]. Wander et al. studied the relationship between HGS and occurrence of diabetes among Japanese-Americans who had not been diagnosed with diabetes in the United States and found an inverse association between HGS and the incidence of diabetes in the leaner group with a low BMI [10]. However, the correlation was significant only in the low-BMI group. Moreover, the number of participants included was relatively low (approximately 390) [10]. Therefore, we conducted this population-based prospective cohort study to investigate the relationship between RGS and newly diagnosed diabetes.

## 2. Materials and Methods

### 2.1. Study Population

Data for our study were obtained from the Korean Genome and Epidemiology Study (KoGES) of the general Korean population. Our study utilized data specifically from the KoGES_Health Examinee (HEXA) study, which recruited subjects aged ≥ 40 years at the baseline from various clinics. This population-based prospective cohort study aimed to investigate the effect of environmental and lifestyle factors on chronic diseases such as metabolic syndrome, obesity, hypertension, diabetes, osteoporosis, and chronic kidney disease (CKD). Detailed information regarding the study design and data collection methods for KoGES has been described previously [11].

The baseline HEXA study, conducted between 2004 and 2013, included a total of 173,195 men and women aged 40–80 years. Eligible participants were solicited for baseline recruitment through various methods, including on-site invitations, mail invitations, phone calls, or media campaigns. The responding individuals were asked to come to health centers to fill out a questionnaire managed by trained staff, to undergo a physical examination, and to provide blood sampling. This study was conducted at 38 health centers nationwide. A follow-up study was subsequently conducted between 2007 and 2016. Participants in the follow-up study were periodically asked to fill out surveys, which were sent by mail and discussed through phone calls.

**Figure 1:**
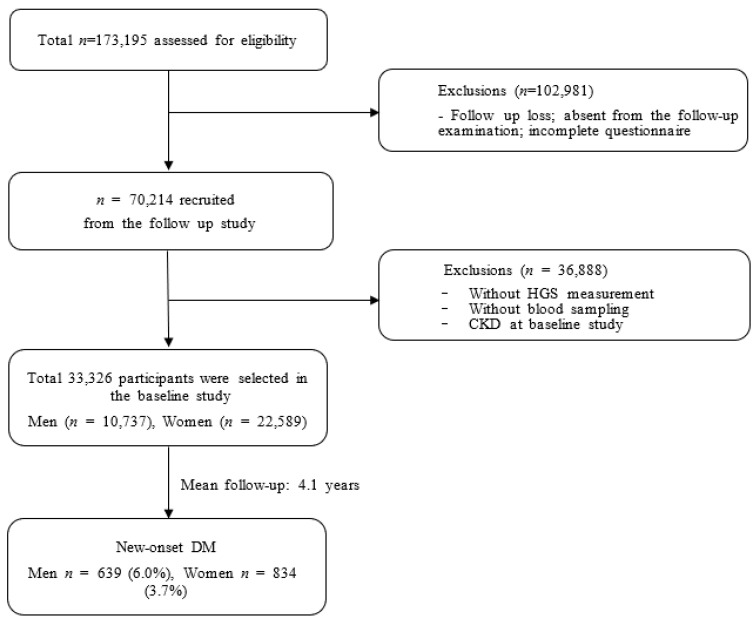
Flow diagram representing studies that meet inclusion/exclusion criteria.

In our analysis, we excluded participants from the baseline study who met any of the following criteria: (1) follow-up loss, (2) absence of HGS data, (3) absence of laboratory data, or (4) diagnosed with DM during the baseline study (Figure 1).

### 2.2. Measurement of Handgrip Strength

HGS measurement was performed twice with a one-minute interval by squeezing a digital handgrip strength dynamometer (T.K.K. 5401, TAKEI Scientific Instruments Co., Ltd., Nigata, Japan) [12]. The participants were instructed to apply maximum force on the dynamometer. HGS was measured once the grip was sustained at an angle of 15° from the flexion of the hip. Absolute HGS was regarded as the highest value derived from both hands and denoted in kg [13]. In order to minimize the influence of body size on HGS, RGS was chosen. RGS was calculated by dividing the absolute HGS by BMI, which has been previously used as a predictor of muscle strength [14]. RGS data were segmented into quartiles according to sex.

### 2.3. Anthropometric and Laboratory Measurements and General Data

Data on anthropometry, demographics, lifestyle, and laboratory tests were comprehensively obtained. The anthropometric data consisted of sex, age, waist circumference, BMI, systolic blood pressure (SBP), and diastolic blood pressure (DBP). Waist circumference was measured with a flexible tape (Seca 220; Seca GmBH & Co Kg, Hamburg, Germany) positioned at the midpoint between the lower edge of the rib and the upper border of the iliac crest during exhalation [15]. BMI was defined as weight divided by height squared (kg/m^2^). Blood pressure (BP) was collected with a mercury sphygmomanometer after a 5 min rest period in a seated position (Baumanometer Wall Unit 33 [0850]). All BP measurements were conducted twice on the right arm at 30 s intervals using the same instrument [16]. Participants were diagnosed with hypertension if their SBP was ≥140 mmHg, DBP was ≥90 mmHg, or if they were on antihypertensive medications [17]. Medication history was collected by administering questionnaires. Besides medication history, the participants also provided information regarding demographics, lifestyle habits, and medical conditions via questionnaires. These included sex, age, alcohol intake, smoking history, regular exercise habits, along with current and past medical histories. Alcohol consumption data were gathered through the questionnaire, which detailed the type (hard liquor, soju, or beer), quantity, and frequency of alcohol consumption. Alcohol intake was classified as consuming alcohol at least once per week, with the cutoff values for weekly alcohol intake set at >140 g for men and >70 g for women [18]. Smoking status categories included current smokers who confirmed that they had smoked more than five packs of cigarettes in their lifetime and still smoked, ex-smokers who had smoked more than five packs but no longer smoked, and never smokers who had smoked less than five packs in their lifetime [19]. Regular exercise was classified as engaging in intense physical activity at least three times per week. Patients with cardiovascular disease were identified as those who responded “yes” to the statement “I have been diagnosed with cardiovascular disease by a physician”. The Global Physical Activity Questionnaire (GPAQ) was used to evaluate the physical activity levels [20]. Laboratory tests were performed to measure aspartate aminotransferase (AST), alanine aminotransferase (ALT), total cholesterol (TC), high-density lipoprotein (HDL) cholesterol, and triglyceride levels using high-performance liquid chromatography with an automated HGLC-723G7 analyzer (Tosoh Corporation, Tokyo, Japan).

### 2.4. Definition of DM

DM was diagnosed if any of the following criteria were met: fasting plasma glucose ≥ 126 mg/dL, glycosylated hemoglobin (HbA1c) ≥ 6.5%, or plasma glucose level 2 h after 75 g oral glucose tolerance test (OGTT) ≥ 200 mg/dL [21]. Participants who confirmed that they were taking medications for diabetes through their questionnaire responses were also considered to have diabetes. The series of medication history was obtained from the questionnaires.

### 2.5. Statistical Analysis

All covariates were subjected to statistical analysis. Chi-square tests were used for categorical variables, while independent *t*-test and analysis of variance (ANOVA) tests were performed for continuous variables. Categorical and continuous variables were shown as *n* (%) and mean ± standard deviation, respectively.

To assess the relationship between RGS and incidence of DM, Cox regression analysis was performed after adjusting for age, alcohol consumption, smoking status, regular exercise, presence of hypertension and cardiovascular disease, SBP, DBP, AST, ALT, TC, HDL-cholesterol, and triglyceride levels.

RGS data were categorized into quartiles: Q1 was ≤1.40; Q2 ranged between 1.40 and 1.61; Q3 ranged between 1.61 and 1.84; and Q4 was >1.84 in men. For women, these values were defined as follows: Q1 was ≤0.85; Q2 ranged between 0.85 and 1.01; Q3 ranged between 1.01 and 1.17; and Q4 was >1.17. The group with the lowest RGS value (Q1) served as the reference group in our study. Cox regression analysis was also used to compute the hazard ratios (HRs) and 95% confidence intervals (CIs) of new-onset DM for RGS quartiles after adjusting for potential confounders.

Kaplan–Meier curves were plotted to examine the cumulative incidence rate of incident DM according to baseline RGS quartiles. Receiver operating characteristic (ROC) curves were illustrated, and area under the ROC curves were calculated. Furthermore, cut-off values were analyzed, which were defined as the points closest to the upper-left corner. Statistical significance was set at *p*-value < 0.05. Statistical analyses were conducted using SPSS version 28.0 (IBM Corp., Armonk, NY, USA).

## 3. Results

Table 1 and Table 2 present the baseline characteristics of the study population categorized by baseline RGS quartiles. The study included a total of 33,326 participants (10,737 men and 22,589 women). A significant decrease in the mean values of some covariates were observed with increasing RGS quartiles. These variables included age, waist circumference, BMI, ALT, SBP, DBP, hypertension, and cardiovascular disease in men. In women, the variables included age, waist circumference, BMI, total cholesterol, triglycerides, AST, ALT, SBP, DBP, hypertension, and cardiovascular disease. HDL-cholesterol levels were found to increase with increasing RGS quartiles in both men and women.

**Figure 2:**
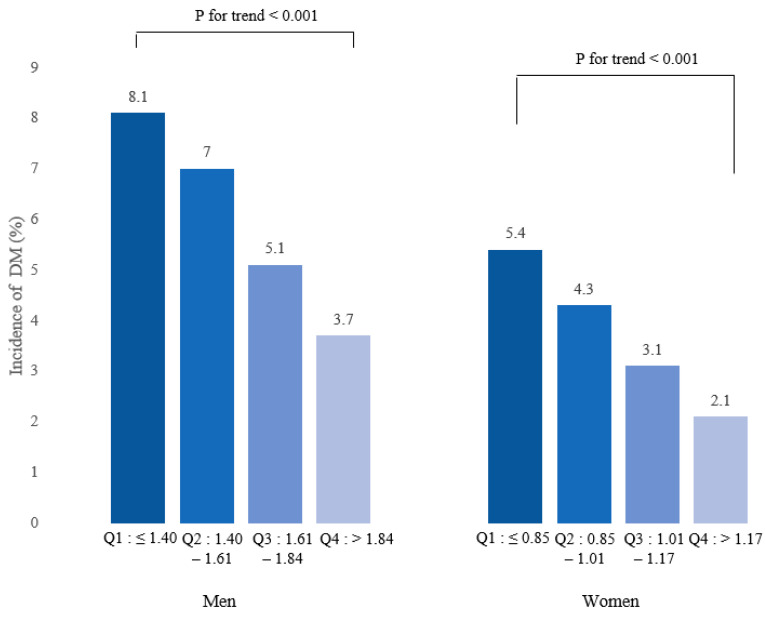
Incidence of DM according to baseline RGS quartiles.

A decline in the incidence of DM was observed with the increase in baseline RGS quartiles for both men and women (Figure 2). These findings show that a dose–response association exists between RGS and DM. The results of the association of baseline RGS with the new onset of DM in Koreans are summarized in Table 3. RGS was negatively associated with incident DM in all models for both men and women.

Table 4 suggests the HRs and 95% CIs for incidence of DM based on baseline RGS quartile. The lowest RGS quartile (Q1) was designated as the reference group [14]. In comparison to the reference group and after adjustments were made in model 3, the HRs (95% CI) for DM of the participants were 0.72 (0.54–0.95) for the Q3 group of men, 0.60 (0.43–0.84) for the Q4 group of men, and 0.72 (0.52–0.99) for the Q4 group of women, which were statistically significant.

**Figure 3:**
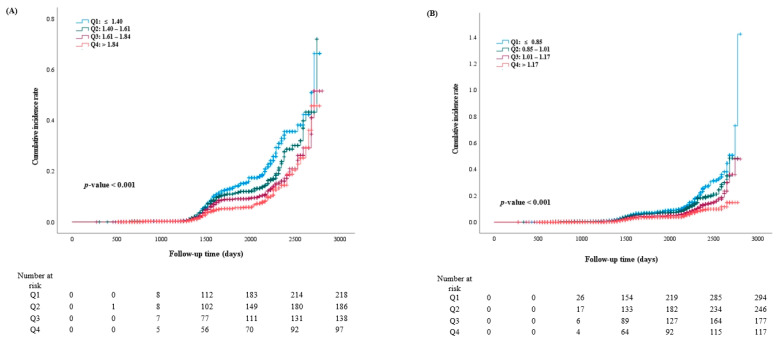
Kaplan–Meier curve for incident DM according to baseline RGS quartile in men (**A**) and in women (**B**).

**Figure 4:**
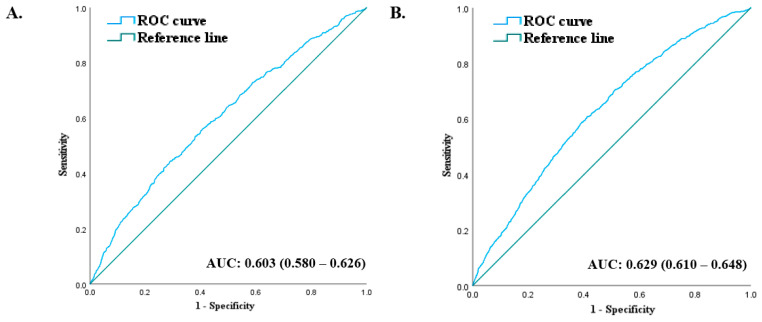
ROC curve presenting the predictive power for incident DM according to baseline RGS in men (**A**) and in women (**B**).

Throughout the 90-month follow-up period, incident DM occurred in 1473 individuals (4.4%, 1473/33,326). The Q1 group had the highest cumulative incident rates of newly developed DM during the 90 months of follow-up. However, these rates gradually declined from Q2 to Q4 in both men and women after the baseline survey (log-rank test, *p* < 0.001) (Figure 3). ROC curves were plotted to confirm whether RGS could be used as an indicator of DM (Figure 4). The area under the curve (AUC) in Figure 4A was 0.603 (0.580–0.626), with an optimal cut-off value of 1.64. The AUC in Figure 4B was 0.629 (0.610–0.648), with an optimal cut-off value of 1.01.

## 4. Discussion

This study investigated the relationship between sarcopenia, represented by RGS, and diabetes. The results showed that the incidence of diabetes decreased with the increase in RGS. Our study found a correlation between RGS and incident diabetes.

Even after adjusting for diabetes risk factors such as age, regular exercise, alcohol intake, and smoking status, the dose-dependent relationship between RGS and new-onset diabetes was statistically significant. Therefore, regular assessment of RGS could help in the prevention or early diagnosis of diabetes. Several studies have previously suggested that handgrip strength was associated with physical activity [22,23,24]. Various kinds of exercise could improve handgrip strength. Based on our findings, interventions such as diet control, muscle exercise, and arrangement of follow-up periods can be implemented. In other words, clinicians can recommend exercise to patients and check the effect of exercise by assessing RGS.

The findings of our study are related to those from previous reports in terms of the relationship between HGS and fasting glucose levels [25], HGS and prediabetes prevalence [26], and HGS and diabetes incidence [27]. However, the majority of the previous studies were cross-sectional and examined the relationship between HGS and the prevalence of diabetes. Moreover, studies on the incidence of diabetes had relatively small sample sizes. Our study has the advantage of uncovering the relationship between RGS and diabetes incidence across a large number of population-based prospective cohort studies. Additionally, even after adjusting for age, cardiovascular disease, hypertension, regular exercise, smoking status, alcohol intake BMI, SBP, DBP, AST, ALT, total cholesterol, HDL-cholesterol, and triglyceride levels, the risk of diabetes was found to be higher in the low-RGS group than in the high-RGS group.

The mechanism that can explain the relationship between RGS and diabetes incidence involves the GLUT-4 glucose transporter. It is well known that glucose enters the muscle cells through the GLUT-4 glucose transporter. Exercise is the most powerful factor that increases GLUT-4 expression in skeletal muscles [28]. In other words, muscle strengthening enhances insulin activity, glucose consumption, and muscle glycogen storage, thereby decreasing the incidence of diabetes.

Another potential mechanism associated with HGS and diabetes can be confirmed through the relevant mediators. Sarcopenia is related to inflammation, a crucial element in insulin resistance [29]. A decrease in muscle strength is associated with a rise in inflammatory markers such as tumor necrosis factor-alpha (TNF-alpha), interleukin-6 (IL-6), and c-reactive protein (CRP), which can trigger the incidence of diabetes [30,31]. Several studies have reported the inverse association of IL-6 and TNF-alpha with muscle mass [32,33]. In addition, inflammatory markers, such as CRP and IL-6, can be predictors of incident type 2 DM [34]. These markers are significantly associated with endothelial vasoreactivity, as they reduce the response to vasodilatory agents [35]. In other words, inflammation can alter endothelial permeability and decrease peripheral blood flow, thereby limiting insulin delivery and promoting insulin resistance [36]. Furthermore, oxidative stress which can induce inflammation is associated with diabetes [37]. There exists a strong correlation between inflammation and oxidative stress. The immune system activates ROS (reactive oxygen species)-producing macrophages to eliminate pathogens. The accumulation of ROS triggers the activation of mitochondrial uncoupling protein-2 (UCP-2), which decreases ATP production. This starts a series of reactions that eventually lead to the impairment of insulin secreted from pancreatic beta cells [38]. On the other hand, physical activity is broadly advocated due to of its many health advantages, encompassing enhancements in heart and pulmonary function, body composition, and sugar control [39]. It has been recently suggested that physical exercise prompts the generation of ROS due to increased muscle activity, causing a transient imbalance [40]. Nevertheless, the production of ROS during exercise is temporary [40]. On the contrary, antioxidants (i.e., superoxide dismutase, catalase, glutathione, nuclear factor erythroid 2-related factor 2 and glutathione peroxidase) are released during exercise, which in turn enhances the antioxidant defense system and lowers concentrations of oxidative stress biomarkers [41]. The relationship between RGS and incident diabetes can be explained through the mechanism.

Despite recruiting a large number of participants, our study has several limitations that need consideration. First, type 1 and 2 diabetes could not be distinguished. The KoGES data did not contain information regarding serum insulin, C-peptide, and pancreatic autoantibodies that could be used to diagnose type 1 diabetes. Second, the incidence of diabetes is likely to decrease with the increase in muscle mass, strength, and function. However, in our study and a previous study [42], a significant relationship was obtained between HGS and diabetes incidence, even after adjusting for regular exercise. This may be due to the fact that regular exercise evaluation through questionnaire surveys has difficulty quantifying the type and time of exercise. Moreover, regular exercise does not immediately lead to an increase in HGS. Third, family history, an important risk factor for diabetes, was not included due to a shortage of data. Consequently, the influence of genetics on diabetes development was not confirmed. Furthermore, several diseases that can affect muscle strength such as muscular disorders, COPD, and heart failure were not available in the cohort data. Finally, an adequate index excluding the effect of body size (height, weight, and BMI) on HGS strength was not determined. Nevertheless, RGS was used to reduce the impact of body size [43]. Additional studies are required to determine indices which are independent of body size.

## 5. Conclusions

Baseline RGS predicted incident diabetes among adults in Korea. These findings suggest that RGS may be used to identify individuals at high risk of diabetes who may benefit from early intervention. This would aid in reducing the risk of diabetes.

## Figures and Tables

**Table 1:** Baseline characteristics of the study population according to baseline RGS quartile in men.

	Men	Q1	Q2	Q3	Q4	*p*-Value
		≤1.40	1.40–1.61	1.61–1.84	>1.84	
*n*	10,737	2693	2675	2715	2654	
HGS (kg)	39.1 ± 8.5	30.1 ± 6.2	37.3 ± 37.3	41.4 ± 4.3	47.5 ± 7.6	<0.001
RGS (kg/BMI)	1.62 ± 0.37	1.18 ± 0.2	1.51 ± 0.06	1.72 ± 0.07	2.08 ± 0.26	<0.001
Age (years)	54.8 ± 8.5	57.9 ± 8.1	55.9 ± 8.1	54.2 ± 8.2	50.9 ± 7.9	<0.001
Waist circumference (cm)	85.1 ± 7.4	88.0 ± 7.4	86.0 ± 6.7	84.6 ± 6.9	81.4 ± 7.0	<0.001
BMI (kg/m^2^)	24.3 ± 2.7	25.6 ± 2.8	24.8 ± 2.4	24.1 ± 2.4	22.8 ± 2.4	<0.001
Total cholesterol (mg/dL)	193.6 ± 33.9	193.9 ± 34.5	195.0 ± 34.4	193.9 ± 33.8	191.7 ± 33.0	0.004
HDL-cholesterol (mg/dL)	50.0 ± 12.0	48.4 ± 11.3	49.4 ± 11.9	50.3 ± 11.9	52.0 ± 12.6	<0.001
Triglyceride (mg/dL)	145.5 ± 96.1	150.5 ± 92.5	151.0 ± 98.2	145.4 ± 93.6	135.0 ± 99.4	<0.001
AST (IU/L)	24.8 ± 12.9	25.2 ± 11.5	25.5 ± 15.8	24.8 ± 13.1	23.8 ± 10.5	<0.001
ALT (IU/L)	25.4 ± 18.2	26.7 ± 16.5	26.7 ± 24.6	25.2 ± 15.6	22.9 ± 13.8	<0.001
SBP (mmHg)	125.3 ± 13.8	126.9 ± 14.1	125.6 ± 13.7	125.3 ± 13.7	123.5 ± 13.6	<0.001
DBP (mmHg)	78.1 ± 9.4	79.0 ± 9.3	78.2 ± 9.30	78.0 ± 9.4	77.2 ± 9.5	<0.001
Alcohol intake, *n* (%)	3662 (34.1)	803 (29.8)	896 (33.5)	982 (36.2)	981 (37.0)	<0.001
Smoking status, *n* (%)						<0.001
Never smoker	2903 (27.1)	782 (29.1)	719 (27.0)	740 (27.3)	662 (25.0)	
Ex-smoker	4828 (45.1)	1266 (47.2)	1229 (46.1)	1236 (45.7)	1097 (41.5)	
Current smoker	2968 (27.7)	635 (23.7)	716 (26.9)	730 (27.0)	887 (33.5)	
Regular exercise, *n* (%)	4467 (41.6)	1141 (42.4)	1160 (43.4)	1570 (42.2)	1633 (38.5)	0.002
Hypertension, *n* (%)	2402 (22.4)	872 (32.4)	651 (24.4)	552 (20.4)	327 (12.3)	<0.001
CVD, *n* (%)	395 (3.7)	136 (5.1)	114 (4.3)	89 (3.3)	56 (2.1)	<0.001

HGS, handgrip strength; RGS, relative handgrip strength; BMI, body mass index; HDL, high-density lipoprotein; AST, aspartate aminotransferase; ALT, alanine aminotransferase; SBP, systolic blood pressure; DBP, diastolic blood pressure; CVD, cardiovascular disease.

**Table 2:** Baseline characteristics of the study population according to baseline RGS quartile in women.

	Women	Q1	Q2	Q3	Q4	*p*-Value
		≤0.85	0.85–1.01	1.01–1.17	>1.17	
*n*	22,589	5492	5752	5717	5628	
HGS (kg)	23.5 ± 2.2	17.9 ± 3.7	22.4 ± 2.5	25.0 ± 2.6	28.7 ± 4.6	<0.001
RGS (kg/BMI)	1.02 ± 0.25	0.71 ± 0.13	0.93 ± 0.05	1.09 ± 0.05	1.33 ± 0.18	<0.001
Age (years)	52.9 ± 7.7	56.5 ± 7.5	54.0 ± 7.4	51.9 ± 7.2	49.3 ± 6.8	<0.001
Waist circumference (cm)	77.5 ± 8.0	81.7 ± 8.2	78.8 ± 7.5	76.5 ± 7.1	73.2 ± 6.7	<0.001
BMI (kg/m^2^)	23.5 ± 2.9	25.3 ± 3.1	24.0 ± 2.6	23.0 ± 2.3	21.6 ± 2.2	<0.001
Total cholesterol (mg/dL)	200.2 ± 35.0	204.2 ± 36.3	202.3 ± 35.1	199.6 ± 34.8	194.9 ± 33.3	<0.001
HDL-cholesterol (mg/dL)	56.7 ± 13.0	54.3 ± 12.3	55.8 ± 12.6	57.0 ± 12.9	59.8 ± 13.5	<0.001
Triglyceride (mg/dL)	111.0 ± 70.0	124.2 ± 73.9	116.3 ± 76.4	108.3 ± 66.0	95.2 ± 58.9	<0.001
AST (IU/L)	22.0 ± 9.9	23.1 ± 10.5	22.4 ± 12.4	21.7 ± 8.8	20.8 ± 7.0	<0.001
ALT (IU/L)	19.0 ± 14.6	21.2 ± 17.8	19.7 ± 17.0	18.4 ± 11.1	16.8 ± 10.6	<0.001
SBP (mmHg)	120.6 ± 14.6	122.7 ± 14.6	121.4 ± 14.7	120.3 ± 14.7	117.8 ± 14.1	<0.001
DBP (mmHg)	74.1 ± 9.4	75.3 ± 9.3	74.5 ± 9.4	73.9 ± 9.5	72.6 ± 9.3	<0.001
Alcohol intake, *n* (%)	1117 (4.9)	206 (3.8)	267 (4.6)	306 (5.4)	338 (6.0)	<0.001
Smoking status, *n* (%)						0.019
Never smoker	21,774 (96.9)	5314 (97.2)	5536 (96.9)	5527 (97.3)	5397 (96.3)	
Ex-smoker	289 (1.3)	73 (1.3)	71 (1.2)	66 (0.2)	79 (1.4)	
Current smoker	405 (1.8)	81 (1.5)	105 (1.8)	89 (1.6)	130 (2.3)	
Regular exercise, *n* (%)	9386 (41.6)	3359 (38.8)	2368 (41.2)	2477 (43.3)	2408 (42.8)	<0.001
Hypertension, *n* (%)	3592 (15.9)	1290 (23.5)	1019 (17.8)	796 (13.9)	487 (8.7)	<0.001
CVD, *n* (%)	439 (1.9)	199 (3.6)	120 (2.1)	84 (1.5)	36 (0.6)	<0.001

HGS, handgrip strength; RGS, relative handgrip strength; BMI, body mass index; HDL, high-density lipoprotein; AST, aspartate aminotransferase; ALT, alanine aminotransferase; SBP, systolic blood pressure; DBP, diastolic blood pressure; CVD, cardiovascular disease.

**Table 3:** Association between baseline RGS (per 0.01 kg) and incidence of DM in Koreans using Cox regression analysis.

Men			Women		
	HR	*p*-Value		HR	*p*-Value
Unadjusted	0.52 (0.42–0.65)	<0.001	Unadjusted	0.27 (0.20–0.36)	<0.001
Model 1	0.57 (0.45–0.72)	<0.001	Model 1	0.43 (0.31–0.58)	<0.001
Model 2	0.55 (0.40–0.74)	<0.001	Model 2	0.43 (0.28–0.67)	<0.001
Model 3	0.62 (0.45–0.85)	0.003	Model 3	0.61 (0.39–0.95)	0.029

RGS, relative handgrip strength; DM, diabetes mellitus; HR, hazard ratio. Model 1: adjusted for age; Model 2: adjusted for age, regular exercise, alcohol intake, and smoking status; Model 3: adjusted for age, hypertension, cardiovascular disease, regular exercise, alcohol intake, smoking status, SBP, DBP, AST, ALT, total cholesterol, HDL-cholesterol, and triglycerides.

**Table 4:** Hazard ratio and 95% confidence intervals for incident DM according to baseline RGS quartile.

	Men				Women			
	Q_1_	Q_2_	Q_3_	Q_4_	Q_1_	Q_2_	Q_3_	Q_4_
	≤1.40	1.40–1.61	1.61–1.84	>1.84	≤0.85	0.85–1.01	1.07–1.17	>1.17
*n*	2693	2675	2715	2654	5492	5752	5717	5628
Unadjusted	1.00	0.84 (0.69–1.02)	0.64 (0.51–0.79)	0.49 (0.39–0.63)	1.00	0.82 (0.69–0.97)	0.57 (0.48–0.69)	0.41 (0.33–0.51)
Model 1	1.00	0.86 (0.71–1.05)	0.67 (0.54–0.83)	0.54 (0.42–0.69)	1.00	0.91 (0.76–1.08)	0.70 (0.58–0.84)	0.56 (0.45–0.71)
Model 2	1.00	0.78 (0.61–1.01)	0.68 (0.51–0.89)	0.52 (0.38–0.72)	1.00	0.95 (0.75–1.22)	0.74 (0.57–0.97)	0.56 (0.41–0.77)
Model 3	1.00	0.82 (0.63–1.06)	0.72 (0.54–0.95)	0.60 (0.43–0.84)	1.00	1.03 (0.80–1.32)	0.82 (0.63–1.08)	0.72 (0.52–0.99)

Model 1: adjusted for age; Model 2: adjusted for age, regular exercise, alcohol intake, and smoking status; Model 3: adjusted for age, hypertension, cardiovascular disease, regular exercise, alcohol intake, smoking status, SBP, DBP, AST, ALT, total cholesterol, HDL-cholesterol, and triglycerides.

## Data Availability

Data used in this study were obtained from the Korean Genome and Epidemiology Study (KoGES; 4851-302). These data are available through an online sharing service with permission from the Division of Epidemiology and Health Index in the Korea Centres for Disease Control and Prevention (KCDC) at http://www.kdca.go.kr/contents/ (accessed on 8 December 2022).
